# Elastic Properties of Single-Walled Phosphide Nanotubes: Numerical Simulation Study

**DOI:** 10.3390/nano12142360

**Published:** 2022-07-10

**Authors:** Nataliya A. Sakharova, Jorge M. Antunes, André F. G. Pereira, Bruno M. Chaparro, José V. Fernandes

**Affiliations:** 1 Centre for Mechanical Engineering, Materials and Processes (CEMMPRE), Department of Mechanical Engineering, University of Coimbra, Rua Luís Reis Santos, Pinhal de Marrocos, 3030-788 Coimbra, Portugal; jorge.antunes@dem.uc.pt (J.M.A.); andre.pereira@uc.pt (A.F.G.P.); valdemar.fernandes@dem.uc.pt (J.V.F.); 2 Abrantes High School of Technology, Polytechnic Institute of Tomar, Quinta do Contador, Estrada da Serra, 2300-313 Tomar, Portugal; bruno.chaparro@ipt.pt

**Keywords:** non-carbon nanotubes, numerical simulation, force constants, rigidity, elastic moduli

## Abstract

After a large-scale investigation into carbon nanotubes, significant research efforts have been devoted to discovering and synthesizing other nanotubes formed by chemical elements other than carbon. Among them, non-carbon nanotubes based on compounds of the elements of the 13th group of the periodic table and phosphorus. These inorganic nanotubes have proved to be more suitable candidates than carbon nanotubes for the construction of novel electronic and optical-electronic nano-devices. For this reason, until recently, mainly the structural and electrical properties of phosphide nanotubes were investigated, and studies to understand their mechanical behavior are infrequent. In the present work, the elastic properties of single-walled boron phosphide, aluminum phosphide, gallium phosphide and indium phosphide nanotubes were numerically evaluated using a nanoscale continuum modelling (also called molecular structural mechanics) approach. The force field constants required to assess the input parameters for numerical simulations were calculated for boron phosphide, aluminum phosphide, gallium phosphide and indium phosphide nanostructures using two different methods. The influence of input parameters on the elastic properties evaluated by numerical simulation was studied. A robust methodology to calculate the surface elastic moduli of phosphide nanotubes is proposed.

## 1. Introduction

Achievements in the fabrication of carbon nanotubes (CNTs) and their successful employing in numerous applications have given impetus to prediction and synthesis of new one-dimensional (1D) tubular structures with a honeycomb atomic arrangement. These nanostructures with graphene-like hexagonal lattice are based on compounds of chemical elements other than carbon. Among the examples of such compounds are those based on the elements of the 13th group of the periodic table, such as boron (B), aluminum (Al), gallium (Ga) and indium (In), which are able to establish with phosphorus (P) honeycomb diatomic arrangements, forming nanotubes (NTs) of boron phosphide (BP), aluminum phosphide (AlP), gallium phosphide (GaP) and indium phosphide (InP). These phosphorus-based diatomic NTs are, in general, broadband gap semiconductors with valuable electronics and optoelectronics properties and have promising applications as light-emitting devices operating in the visible range, such as light-emitting diodes (LEDs) [1,2,3,4], solar cells [5], building blocks for nano-integrated circuits [6,7] and parts in quantum electronic devices [8,9].

The synthesis of tubular phosphide nanostructures has been a challenge so far. Bakkers and Verheijen [8] synthesized for the first time crystalline InPNTs employing the vapor–liquid–solid (VLS) laser ablation method and not using a template. Yin et al. [10] proposed a simple template-free thermal chemical process in a conventional furnace with controlled reaction temperature and gas flow for synthetizing single-crystalline InPNT. Palit et al. [11], in their recent study, grew single-crystalline InPNTs using the Metal-Organic Chemical Vapor Deposition (MOCVD) method in patterned template developed on germanium substrate by electron beam lithography (ELB) technique. Wu et al. [2] synthesized crystalline GaPNTs using a chemical reaction in a high-temperature tubular furnace and suggested that the growth of the GaP nanotube occurs according to the VLS mechanism [12].

The remaining phosphide nanotubes, BPNTs and AlPNTs, have not yet been synthesized, but these nanotubes were theoretically predicted. Mirzaei and Giahi [13], and Mirzaei and Meskinfam [14] used density functional theory (DFT) calculations to study optimized geometry and characterize the electronic structure of BPNTs. Theoretical results were also obtained regarding the functionalization of single-walled BPNTs and their suitability for drug carriage, using DFT calculations, in two works by Zahra Sayyad-Alangi et al. [15,16]. Lisenkov et al. [17] calculated the equilibrium geometry and energetic stability of AlPNTs, employing DFT, and Mirzaei and Mirzaei [18] examined the electronic structure of AlPNTs also using DFT calculations.

It is worth mentioning that the characterization of structural and electronic properties of GaPNTs and InPNTs was also carried out. Mirzaei and Mirzaei [19] used DFT calculations to optimize structure of single-walled GaPNTs and to compute the electric field gradient (EFG) tensors for the optimized nanotubes structure. Kamal et al. [20] studied the geometric and electronic structures of single-walled GaPNTs, based on ab initio calculations. Srivastava et al. [21] also employed the ab initio method to investigate the stability, electronic band structure and transport properties of single-walled zigzag GaPNTs. The band structure of the InPNTs was studied by Palit et al. [11], using a theoretical model based on the experimental observation of spectral emission lines. Erkoç [22] computed the optimized geometry and electronic properties of single-walled armchair and zigzag InPNTs, using semi-empirical calculations. In their work, Muhsen et al. [23] employed DFT to analyze the stability and electronic properties of single-walled zigzag InPNTs.

As it is of great importance to investigate the prospective employment of phosphide nanotubes in nano-electronics, their structure and electronic properties have been the focus of the research attention so far [18,19,20,21,22,23,24,25]. Consequently, the mechanical properties of the phosphide NTs have been less studied, although the understanding of the NTs’ mechanical behavior can ensure the robustness and appropriate functioning of nano-devices involving nanotubes as components (building blocks).

Until now, the mechanical properties of phosphide NTs have been evaluated in the works of Kochaev [26], and Jiang and Guo [27], both studies exploring theoretical approaches to this end. Kochaev [26] evaluated the surface Young’s modulus, that is, the product of Young’s modulus by the wall thickness of the nanotubes, and the Poisson’s ratio of AlPNTs and GaPNTs, using ab initio simulation within the atomistic approach. Jiang and Guo [27] applied the “stick-and-spring” model to obtain closed-form analytical solutions for the surface Young’s modulus and Poisson’s ratio and computed these values for BPNTs, GaPNTs and InPNTs. The model of Jiang and Guo [27] was developed under the nanoscale continuum modelling (NCM), also called molecular structural mechanics (MSM), approach, where the bonds between two atoms in the diatomic hexagonal nanostructure are regarded as beams or springs. For successful modelling of these bonds, first, an appropriate choice of the force field constants is necessary, which allows computing of the elastic properties of beams (springs). As for most non-carbon nanotubes, with the exception of boron nitride NTs, the force field constants for phosphide NTs practically do not appear in the literature. To the best of our knowledge, only Jiang and Guo [27] have suggested a method to calculate force constants for BP, GaP and InP nanotubes.

The present study is a systematic evaluation of the elastic properties of single-walled boron phosphide, aluminum phosphide, gallium phosphide and indium phosphide nanotubes (SWBPNTs, SWAlPNTs, SWGaPNTs and SWInPNTs, respectively) in a wide range of chiral indices and diameters, by finite element (FE) simulation. The force field constants for BP, AlP, GaP and InP nanostructures were determined using two different calculation approaches. The influence of the input parameters, selected for the FE modelling and computed basing on two sets of the force field constants, on the elastic properties of phosphide NTs was investigated. A comprehensive analysis was performed on the nanotube wall thickness, required for the calculation of the Young’s and shear moduli. As a result, a robust methodology was proposed to assess the surface Young’s and shear moduli.

## 2. Materials and Methods

### 2.1. Atomic Structure of Phosphide Nanotubes

**Figure 1:**
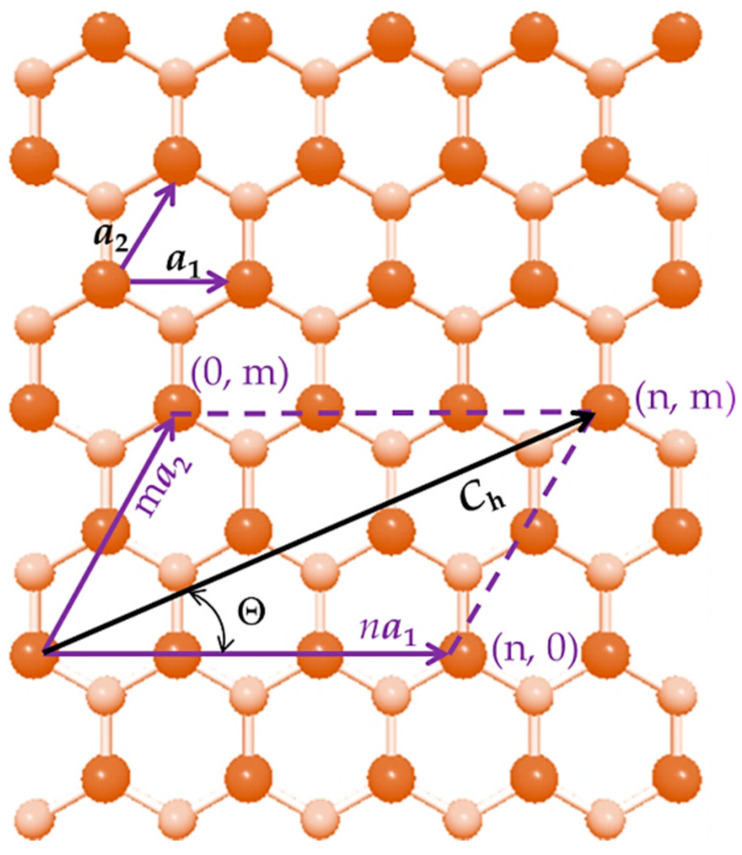
Schematic of the unrolled hexagonal AlP sheet; chiral indices, n and m, chiral vector, ***C_h_***, and chiral angle, Θ, are depicted. P atoms are represented in pale brown; Al atoms are bright brown.

The atomic structure of single-walled phosphide nanotubes is characterized by the chiral vector, ***C_h_***, and the chiral angle, Θ, as follows:

(1) $$C_{h}=na_{1}+ma_{2},$$

(2) $${\Theta =\sin}^{-1}\frac{\sqrt[{}]{3}}{2}\frac{m}{\sqrt[{}]{n^{2}{+\mathrm{nm}+m}^{2}}},$$

where n and m are the chiral indices, both having integers values; $a_{1}$ and $a_{2}$ are the unit vectors of the diatomic hexagonal lattice. This lattice consists of the A13 atom, which is one of the 13th group of the periodic table, such as boron (B), aluminum (Al), gallium (Ga) or indium (In), and phosphorus (P) atom, as shown in Figure 1 for AlP lattice. The length of the unit vector a is expressed by $a=\sqrt{3}a_{A13-P}$, where $a_{A13-P}$ is the equilibrium bond length. As can be seen in Table 1, in which the bond lengths of the phosphide NTs available in the literature are presented, there is no agreement regarding the $a_{A13-P}$ values.

The phosphide NTs are formed by rolling up the respective diatomic hexagonal sheet into a cylinder of diameter, $D_{n}$, which is considered the nanotube diameter and expressed by

(3) $$D_{n}=\frac{a_{A13-P}\sqrt{3\left(n^{2}{+\mathrm{nm}+m}^{2}\right)}}{\pi },$$

where n and m are the chiral indices and $a_{A13-P}$ is the equilibrium bond length of the diatomic structure of the phosphide.

**Figure 2:**
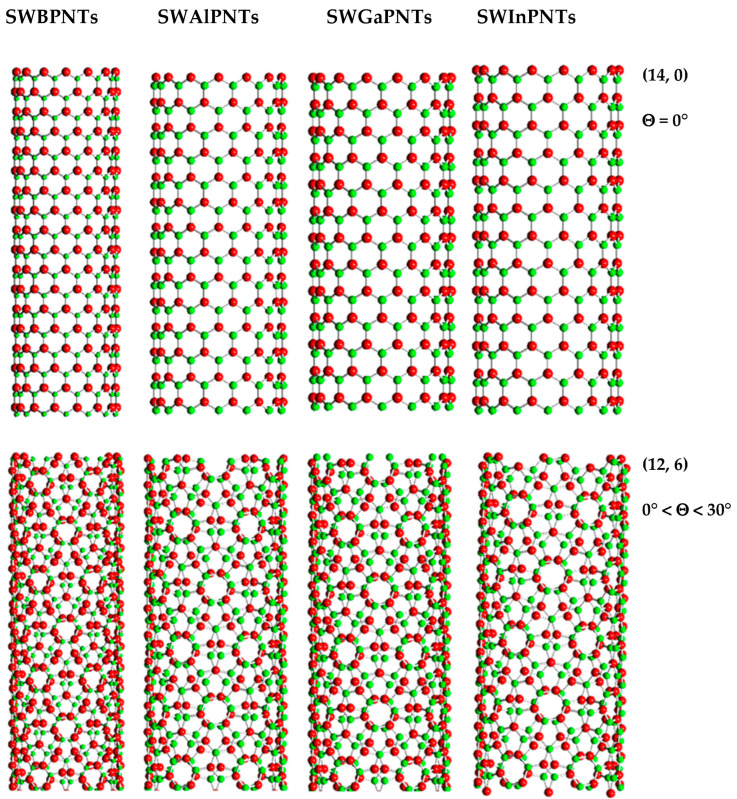

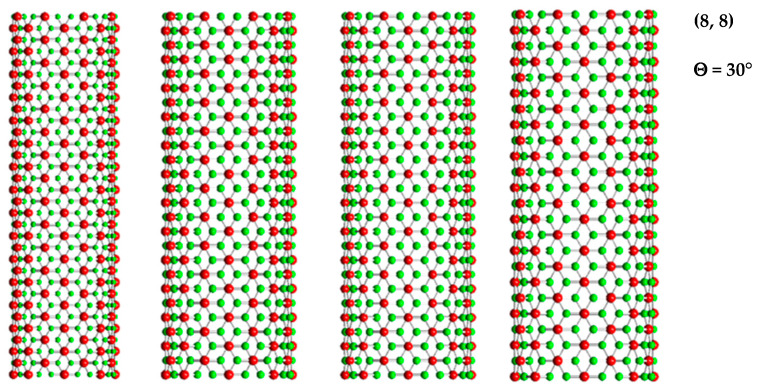
Configurations of (14, 0) zigzag, (12, 6) chiral and (8, 8) armchair of SWBPNTs, SWAlPNTs, SWGaPNTs and SWInPNTs, obtained using the software Nanotube Modeler©. P atoms are in green; B, Al, Ga and In atoms are in red.

The chiral indices, n and m, together with the magnitudes of the chiral angle, which are within the range of 0° to 30° (see Figure 1), allow defining three main symmetry groups of single-walled phosphide nanotubes: for zigzag NTs Θ = 0° and n = 0, for armchair NTs Θ = 30° and n = m, and for chiral NTs 0° < Θ < 30° and n ≠ m ≠ 0. The zigzag and armchair configurations are called non-chiral NTs. Non-chiral (zigzag and armchair) and chiral SWBPNTs, SWAlPNTs, SWGaPNTs and SWInPNTs, displayed in ascending order of the chiral angle, Θ, are represented schematically in Figure 2.

### 2.2. Molecular Mechanics of Phosphide Nanotubes and Equivalent Continuum Properties of Interatomic Bonds

#### 2.2.1. Force Field Constants

According to Mayo et al. [30] and Rappé et al. [31], the total potential energy of a molecular system is presented as the sum of the energy terms due to bonded and non-bonded interactions:

(4) $$U_{\mathrm{total}}{=U}_{\mathrm{bond}}{+U}_{\mathrm{non}-\mathrm{bond}}$$

**Figure 3:**
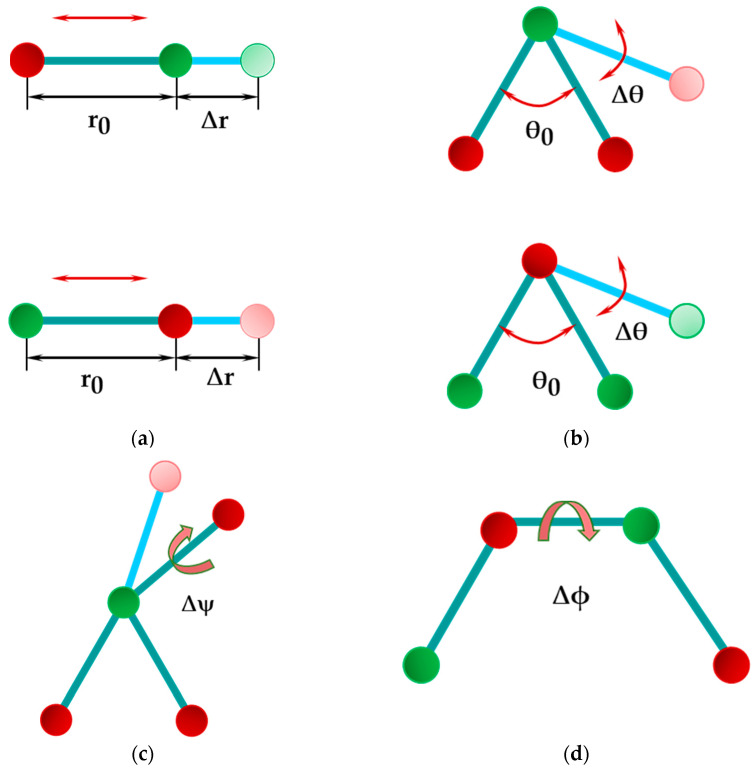
Bond interactions in 13th element phosphide nanostructures: (**a**) bond stretching; (**b**) bond bending; (**c**) dihedral torsion; (**d**) out-of-plane torsion or inversion. P atoms are in green; A13 (B, Al, Ga, In) atoms are in red.

The energy of bonded interactions, which are presented in Figure 3, is expressed through those associated with bond stretching, U_r_, bond bending, U_θ_, dihedral angle torsion, U_ϕ_, and out-of-plane torsion, U_ω_, as follows:

(5) $$U_{\mathrm{bond}}{=U}_{r}{+U}_{\theta }{+U}_{\phi }{+U}_{\omega }.$$

The energy of non-bonded interactions comprises the van der Waals, $U_{\mathrm{vdW}}$, electrostatic, $U_{Q}$, and explicit hydrogen bonds, $U_{\mathrm{Hbs}}$, terms:

(6) $$U_{\mathrm{non}-\mathrm{bond}}{=U}_{\mathrm{vdW}}{+U}_{Q}{+U}_{\mathrm{Hbs}}.$$

In molecular systems, as in the case of phosphide nanotubes, $U_{\mathrm{non}-\mathrm{bond}}$ term can be omitted due to its smallness when compared with $U_{\mathrm{bond}}$ term [27]; thus, $U_{\mathrm{total}}{=U}_{\mathrm{bond}}$. The four terms of Equation (5), which contribute to the total potential energy, $U_{\mathrm{total}}$, are given by the following expressions:

(7) $$U_{r}=\frac{1}{2}k_{r}{\left({r-r}_{0}\right)}^{2},$$

$$U_{\theta }=\frac{1}{2}k_{\theta }{\left({\theta -\theta }_{0}\right)}^{2},$$

$$U_{\phi }=\frac{1}{2}k_{\phi }\left\{1-\cos\left[2\left(\phi -\phi_{0}\right)\right]\right\},$$

$$U_{\psi }=\frac{1}{2}k_{\psi }{\left({\psi -\psi }_{0}\right)}^{2},$$

where $k_{r}$, $k_{\theta }$, $k_{\phi }$ and $k_{\psi }$ are the bond stretching, bond bending, dihedral torsion and inversion force constants, respectively; r, θ, ϕ and ψ are bond length, bond angle, dihedral angle and inversion angle, respectively, and $r_{0}$, $\theta_{0}$, $\phi_{0}$ and $\psi_{0}$ correspond to the equilibrium position (see Figure 3).

The dihedral angle torsion term describes the torsion interaction for two interatomic bonds linked by means of a common bond [30,31] (see Figure 3c). The out-of-plane torsion or inversion term illustrates how difficult it is to rotate three bonds (when one atom in a diatomic honeycomb lattice is bonded to three other atoms), keeping them in the same plane [30] (see Figure 3d). Under the assumption of small deformation and with the approximation that the variation of the dihedral angle, $\left(\phi -\phi_{0}\right)$, is equal to that of the inversion angle, $\left({\psi -\psi }_{0}\right)$, the energies of the dihedral angle torsion and the out-of-plane torsion can be merged into a single equivalent term:

(8) $$U_{\tau }=U_{\phi }+U_{\omega }\Rightarrow$$

$$U_{\tau }=\frac{1}{2}\left({2k}_{\phi }{+k}_{\psi }\right)\cdot {\left(\phi -\phi_{0}\right)}^{2}=\frac{1}{2}k_{\tau }{\left(\phi -\phi_{0}\right)}^{2},$$

where $k_{\tau }$ is the torsional resistance force constant expressed by

(9) $$k_{\tau }={2k}_{\phi }+k_{\psi }.$$

Thus, the total potential energy of the molecular system can be rewritten using three energy terms, $U_{r}$, $U_{\theta }$ and $U_{\tau }$, in the form of harmonic functions, as follows:

(10) $$U_{\mathrm{total}}{=U}_{r}{+U}_{\theta }{+U}_{\tau }=\frac{1}{2}k_{r}{\left(\Delta r\right)}^{2}+\frac{1}{2}k_{\theta }{\left(\Delta \theta \right)}^{2}+\frac{1}{2}k_{\tau }{\left(\Delta \phi \right)}^{2},$$

where Δr, Δθ and Δϕ are the bond stretching increment, bond angle bending variation and angle variation of the twist bond, respectively.

With regard to the calculation of the bond stretching, k_r_, and bond bending, k_θ_, force constants for the diatomic nanostructures, there are two established methods, one based on Universal Force Fields (UFF) [31], and the other using ab initio DFT computations in combination with the analytical expressions obtained using molecular mechanics (MM) [32,33]. Until now, these methods were mainly used for determination of the force field constants of boron nitride nanostructures (see, for example, [32,33,34,35]). Jiang and Guo [27], based on a UFF approach, proposed expressions for k_r_ and k_θ_ force constants for a wide class of non-carbon nanotubes, including BP, GaP and InPNTs.

**Figure 4:**
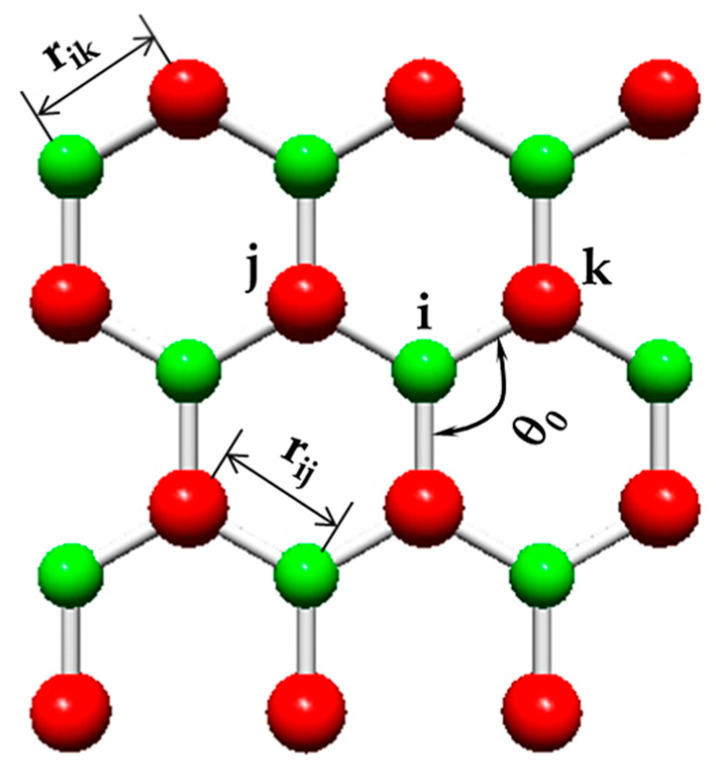
Fragment of the diatomic structure with definition of r_ij_, r_ik_ and θ_0_. P atoms are represented in green and A13 (B, Al, Ga and In) atoms are in red.

According to Rappé et al. [31], the bond stretching constant, $k_{r}$, in the UFF method is determined by the following expression:

(11) $$k_{r}=664.12\frac{Z_{i}^{*}Z_{j}^{*}}{r_{\mathrm{ij}}^{3}},$$

where $Z_{i}^{*}$ and $Z_{j}^{*}$ are the effective charges of the atoms of diatomic nanostructure; $r_{\mathrm{ij}}$ and $r_{\mathrm{ik}}$ are the lengths of the A13–P and P–A13 bonds, respectively, as presented in Figure 4, being $r_{\mathrm{ij}}{=r}_{\mathrm{ik}}{=a}_{A13-P}$ (see Figure 3a).

The bond bending constant, $k_{\theta }$, in the UFF method is determined by the following expression [31]:

(12) $$k_{\theta }=664.12\frac{Z_{i}^{*}Z_{j}^{*}}{r_{\mathrm{jk}}^{5}}\left[{3r}_{\mathrm{ij}}r_{\mathrm{ik}}\left({1-\cos}^{2}\theta_{0}\right){-r}_{\mathrm{jk}}^{2}{\cos\theta }_{0}\right],$$

where $\theta_{0}$ is the angle between neighboring bonds in the diatomic nanostructure (see Figure 4) and $r_{\mathrm{jk}}^{2}{=r}_{\mathrm{ij}}^{2}{+r}_{\mathrm{ik}}^{2}{-2r}_{\mathrm{ij}}r_{\mathrm{ik}}{\cos\theta }_{0}$.

Rappé et al. [31], using UFF, showed that the bond bending constant, k_θ_, of the diatomic nanostructure depends on the three-body angles between the bond pairs A13–P–A13 and P–A13–P (see Figure 3b), which results in two different values for the bond bending constant. Moreover, in the same work, the relationship between two values for the bond bending constant, k_θ1_ and k_θ2_, and the effective charges of the atoms A13 and P ($Z_{1,2}^{*}$) was proposed:

(13) $$\frac{k_{\theta 1}}{k_{\theta 2}}=\frac{Z_{2}^{*2}}{Z_{1}^{*2}}.$$

The alternative method for obtaining the bond stretching and bond bending force constants is based on molecular mechanics (MM) and employs results from DFT calculations. The expressions, which result from MM analytical models for two-dimensional honeycomb diatomic nanostructures, allow relating the surface Young’s modulus, $E_{s}$, and the Poisson’s ratio, ν, with the force field constants, $k_{r},{\;k}_{\theta 1}$ and $k_{\theta 2}$, as follows [32,33]:

(14) $$\{\begin{array}{c} E_{s}=\frac{4\sqrt{3}k_{r}\left(k_{\theta 1}{+k}_{\theta 2}\right)}{k_{r}r_{\mathrm{ij}}^{2}+9\left(k_{\theta 1}{+k}_{\theta 2}\right)}\\ \nu =\frac{k_{r}r_{\mathrm{ij}}^{2}-3\left(k_{\theta 1}{+k}_{\theta 2}\right)}{k_{r}r_{\mathrm{ij}}^{2}+9\left(k_{\theta 1}{+k}_{\theta 2}\right)} \end{array}$$

The values of $E_{s}$ and ν can be obtained experimentally or from ab initio DFT computations (see, for example, [28,36]).

Thus, solving the system of Equations (14) and taking into account Equation (13), the following expressions can be derived to determine the bond stretching, $k_{r}$, and bond bending, $k_{\theta 1}$ and $k_{\theta 2}$, constants of the diatomic nanostructure:

(15) $$k_{r}=\frac{{9E}_{s}}{\sqrt{3}\left(1-\nu \right)},$$

(16) $$k_{\theta 1\left(2\right)}=\frac{E_{s}r_{\mathrm{ij}}^{2}}{\left(1+\frac{Z_{1\left(2\right)}^{*2}}{Z_{2\left(1\right)}^{*2}}\right)\sqrt{3}\left(1+3\nu \right)},$$

where $E_{s}$ and ν are the surface Young’s modulus and Poisson’s ratio of the diatomic sheet, respectively; $Z_{1}^{*}$ and $Z_{2}^{*}$ are the effective charges of the atoms; and $r_{\mathrm{ij}}{=a}_{A13-P}$ is the bond length.

Literature data, which are required to calculate the bond stretching, $k_{r}$, and bond bending, $k_{\theta 1}$ and $k_{\theta 2}$, force constants for phosphide NTs, are summarized in Table 2.

To the best of our knowledge, the methods for calculating the torsional force constant, $k_{\tau }$, for phosphide nanostructures have not been appropriately explored and the values of $k_{\tau }$ have not been reported so far. Jiang and Guo [27] proposed an expression to calculate the inversion force constant, $k_{\psi }$, adopting the UFF method. Among the generic molecular force fields, DREIDING force field [30] allows the determination of the force constants, based only on the hybridization of atoms, regardless of the atoms involved. For diatomic phosphide nanostructures, the DREIDING force field provides the dihedral torsion force constant, $k_{\phi }=25\;\mathrm{kcal}/\mathrm{mol}$, and the inversion force constant, $k_{\psi }=40\;\left(\mathrm{kcal}/\mathrm{mol}\right)/{\mathrm{rad}}^{2}$. Consequently, the torsional resistance force constant, $k_{\tau }$, can already be calculated using Equation (9).

Table 3 presents the bond stretching, $k_{r}$, bond bending, $k_{\theta 1}$ and $k_{\theta 2}$, force constants obtained using both calculation methods, UFF (case 1) and DFT+MM (case 2), and the torsional resistance force constant, $k_{\tau }$, taken from DREIDING, for phosphide nanostructures. As far as we know, there are no values of $E_{s}$ and ν, obtained experimentally or with the help of DFT calculations, which would allow the calculation of bond stretching and bond bending force constants for AlP nanostructures, using Equations (15) and (16). Thus, the study of numerical simulation of the elastic properties of the AlPNTs is limited by case 1 of the set of force constants.

#### 2.2.2. Equivalent Properties of Elastic Beams

**Figure 5:**
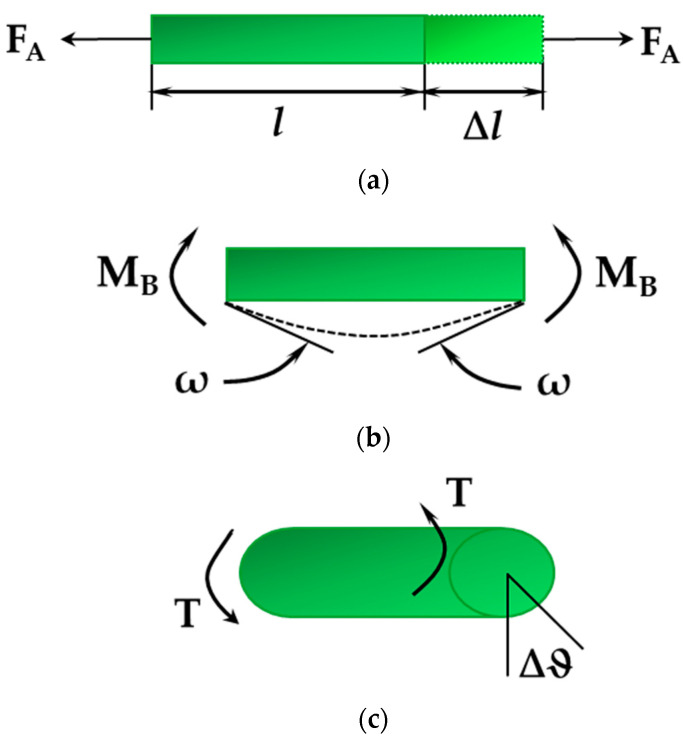
Beam deformation modes: (**a**) pure tension; (**b**) pure bending; (**c**) pure torsion.

In the present study, the NCM/MSM approach was used to evaluate the elastic properties of phosphide NTs. This approach makes use of the connection between the potential energies of bond interactions (see Equation (10) and Figure 3) and the strain energies associated with axial, bending and torsional elastic deformations of equivalent beam elements. Figure 5 shows the beam element undergoing pure tension (a), pure bending (b) and pure torsion (c).

The energies related to stretching, U_A_, bending, U_B_, and torsion, U_T_, of the beam with length, *l*, are expressed as follows:

(17) $$U_{A}=\frac{1}{2}\int_{0}^{L}\frac{F_{A}^{2}}{E_{b}A_{b}}dl=\frac{1}{2}\frac{E_{b}A_{b}}{l}{\left(\Delta l\right)}^{2},$$

(18) $$U_{B}=\frac{1}{2}\int_{0}^{L}\frac{M_{B}^{2}}{E_{b}I_{b}}dl=\frac{1}{2}\frac{E_{b}I_{b}}{l}{\left(2\omega \right)}^{2},$$

(19) $$U_{T}=\frac{1}{2}\int_{0}^{L}\frac{T^{2}}{G_{b}J_{b}}dl=\frac{1}{2}\frac{G_{b}J_{b}}{l}{\left(\Delta \vartheta \right)}^{2},$$

where $F_{A}$ is the axial force, $M_{B}$ is the bending moment, and T is the torsion moment; $E_{b}$ and $G_{b}$ are the beam Young’s and shear moduli, respectively; $A_{b}$ is the beam cross-section area, $I_{b}$ is the beam moment of inertia and $J_{b}$ is the beam polar moment of inertia; $E_{b}A_{b}$ is the beam tensile rigidity, $E_{b}I_{b}$ is the beam bending rigidity and $G_{b}J_{b}$ is the beam torsional rigidity; Δ*l* is the beam axial stretching displacement, ω is the rotational angle at the ends of the beam and Δϑ is the relative rotation between the ends of the beam.

Equations (10) and (17)–(19) allow one to establish the equality between the potential energies related to bond interactions and strain energies associated with elastic deformation of the beam elements, i.e., $U_{r}{=U}_{A}$, $U_{\theta }{=U}_{B}$ and $U_{\tau }{=U}_{T}$. In other words, the tensile, $E_{b}A_{b}$, bending, $E_{b}I_{b}$, and torsional, $G_{b}J_{b}$, rigidities of the beam with length *l* are expressed with the help of bond stretching, $k_{r}$, bond bending, $k_{\theta }$, and torsional resistance, $k_{\tau }$, force constants [37]:

(20) $$E_{b}A_{b}={lk}_{r},$$

(21) $$E_{b}I_{b}={lk}_{\theta },$$

(22) $$G_{b}J_{b}={lk}_{\tau }.$$

Equations (20)–(22) together with the assumption that the beam length, *l,* is equal to the bond length, $a_{A13-P}$, support the use of the NCM/MSM approach to model the mechanical response of phosphide NTs.

Assuming that the beam has a circular cross-section with diameter d, its cross-section area, $A_{b}$, moment of inertia, $I_{b}$, and polar moment of inertia, $J_{b}$, are determined by the following expressions:

(23) $$A_{b}{=\pi d}^{2}/4,$$

(24) $$I_{b}{=\pi d}^{4}/64,$$

(25) $$J_{b}{=\pi d}^{4}/32.$$

The diameter, d, the Young’s modulus, $E_{b}$, and the shear modulus, $G_{b}$, of the beam can be calculated comparing Equations (20)–(22) with Equations (23)–(25) and taking into account the two values of the bond bending constant, $k_{\theta 1}$ and $k_{\theta 2}$, as follows:

(26) $$d=2\sqrt{\frac{2\left(k_{\theta 1}{+k}_{\theta 2}\right)}{k_{r}}},$$

(27) $$E_{b}=\frac{k_{r}^{2}l}{2\pi \left(k_{\theta 1}{+k}_{\theta 2}\right)},$$

(28) $$G_{b}=\frac{k_{r}^{2}k_{\tau }l}{2\pi {\left(k_{\theta 1}{+k}_{\theta 2}\right)}^{2}}.$$

The Poisson’s ratio of the beam can be assessed by the relationship derived from molecular mechanics [32,33,38] as follows:

(29) $$\nu_{b}=\frac{k_{r}l^{2}–\;3\left(k_{\theta 1}{+k}_{\theta 2}\right)}{k_{r}l^{2}+9\left(k_{\theta 1}{+k}_{\theta 2}\right)}.$$

Thus, knowing the values of the force field constants, $k_{r}$, $k_{\theta 1}$ and $k_{\theta 2}$, $k_{\tau }$, for phosphide nanostructures (Table 3), the geometrical and elastic properties of the beam elements can be deduced, as shown in Table 4.

### 2.3. Geometrical Characteristics of Phosphide Nanotubes and FE Analysis

Table 5 shows the geometrical characteristics of SWBPNTs, SWAlPNTs, SWGaPNTs and SWInPNTs of three main configurations, armchair (Θ = 30°), zigzag (Θ = 0°) and chiral (family of Θ = 19.1°), used in the FE analysis. The chiral indices of NTs were chosen in order to obtain structures with comparable diameters. The length of the NTs was chosen to be about 30 times greater than the NT diameter to ensure that the mechanical response of nanotubes is independent of the NT length [39].

The input parameters (two sets of these parameters for each NTs, except for SWAlPNTs) used for FE simulation of phosphide nanotubes are those presented in Table 4.

The meshes of SWBPNTs, SWAlPNTs, SWGaPNTs and SWInPNTs used in FE analyses were constructed with the help of the Nanotube Modeler© software (version 1.8) developed by JCrystalSoft (www.jcrystal.com accessed on 21 June 2022). The in-house application InterfaceNanotubes.NM [39] was used for conversion of the PDB (Program Database) files, obtained from the Nanotube Modeler© software, into the format usable in the ABAQUS^®^ code. Afterwards, the FE code ABAQUS^®^ was used to study the mechanical response of the phosphide NTs under conventional tensile, bending and torsion tests. For this, in order to carry out the respective numerical tests, the axial tensile force, F_a_, the transverse force, F_t_, and the torsional moment, T, were applied to one end of the NT, when the other end was fixed. In the torsion test, the nodes under loading were prevented from moving in the radial direction. The results taken from the FE analysis of the tensile, bending and torsion test are, respectively, the axial displacement, u_a_, the transverse displacement, u_t_, and the twist angle, φ. This makes it possible to determine the tensile, EA, bending, EI, and torsional, GJ, rigidities of the phosphide NT with a length L_n_ by the following expressions:

(30) $$\mathrm{EA}=\frac{F_{a}L_{n}}{u_{a}},$$

(31) $$\mathrm{EI}=\frac{F_{t}L_{n}^{3}}{{3u}_{t}},$$

(32) $$\mathrm{GJ}=\frac{{\mathrm{TL}}_{n}}{\varphi }.$$

## 3. Results and Discussion

### 3.1. Rigidities of SWBPNTs, SWAlPNTs, SWGaPNTs and SWInPNTs

**Figure 6:**
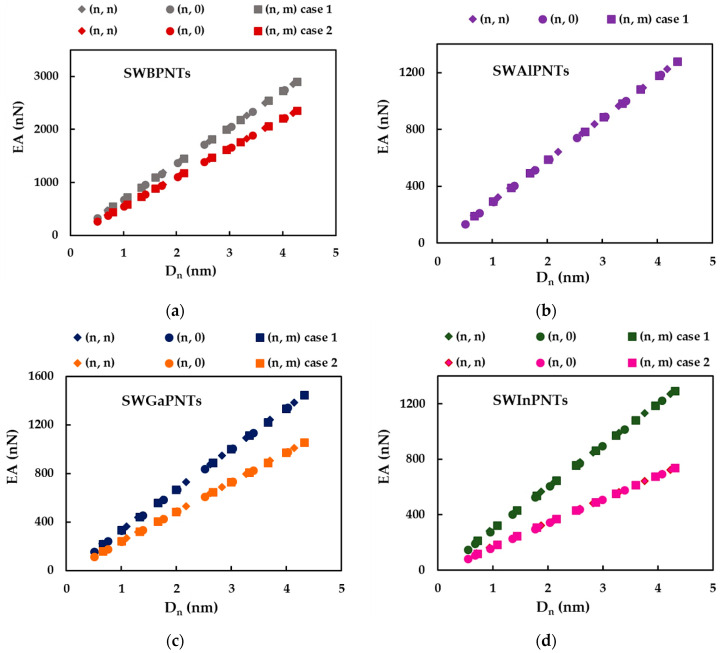
Evolution of tensile rigidity, EA, as a function of the nanotube diameter, $D_{n}$, for: (**a**) SWBPNTs; (**b**) SWAlPNTs; (**c**) SWGaPNTs; and (**d**) SWInPNTs.

**Figure 7:**
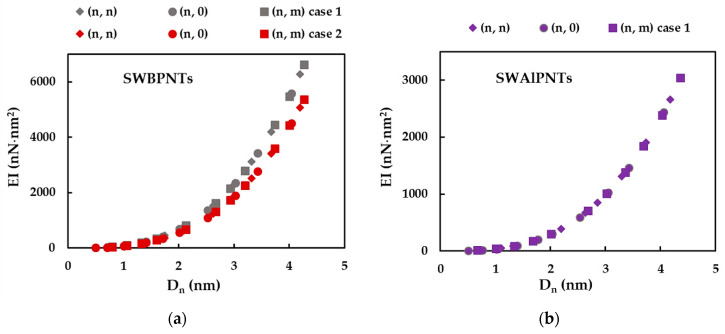

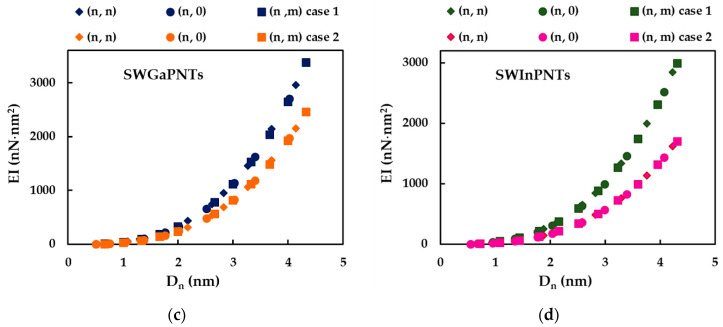
Evolution of bending rigidity, EI, as a function of the nanotube diameter, $D_{n}$, for: (**a**) SWBPNTs; (**b**) SWAlPNTs; (**c**) SWGaPNTs; and (**d**) SWInPNTs.

**Figure 8:**
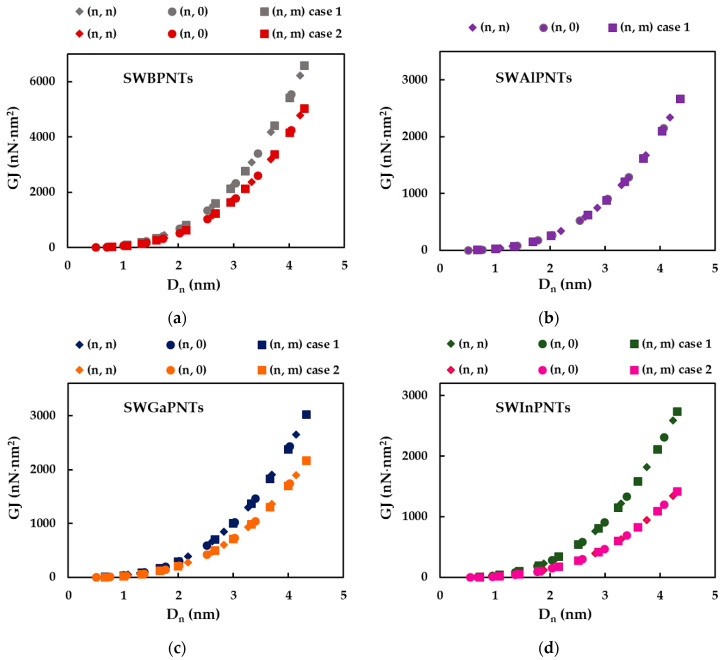
Evolution of torsional rigidity, GJ, as a function of the nanotube diameter, $D_{n}$, for: (**a**) SWBPNTs; (**b**) SWAlPNTs; (**c**) SWGaPNTs; and (**d**) SWInPNTs.

The tensile, EA, bending, EI, and torsional, GJ, rigidities of SWBPNTs, SWAlPNTs, SWGaPNTs and SWInPNTs obtained by Equations (30)–(32) for the two cases (except SWAlPNTs) of the numerical simulation input values presented in Table 4, are plotted as a function of the nanotube diameter, $D_{n}$, in Figure 6, Figure 7 and Figure 8, respectively. For case 1 and case 2 of numerical simulation input parameters from Table 4, the rigidity values for chiral or non-chiral (zigzag and armchair) NTs follow the same trend with increasing $D_{n}$. The EA, EI and GJ rigidities obtained for case 1 (UFF) are higher than those for case 2 (DFT + MM).

**Figure 9:**
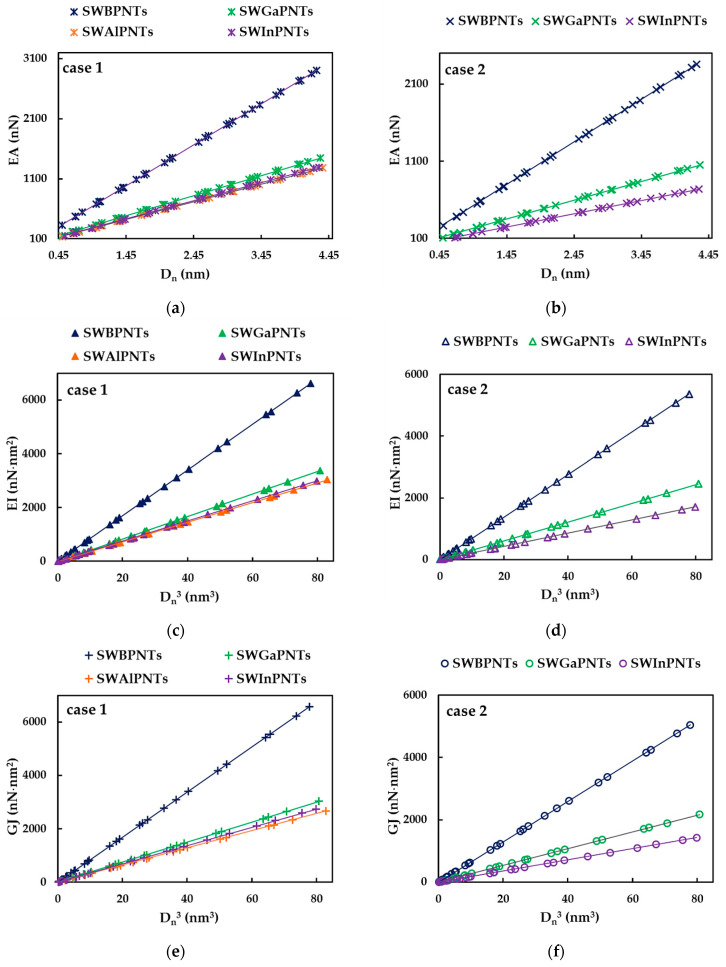
Evolution of rigidities as a function of the nanotube diameter, $D_{n}$, for SWBPNTs, SWAlPNTs, SWGaPNTs and SWInPNTs: EA rigidity for case 1 (**a**) and case 2 (**b**); EI rigidity for case 1 (**c**) and case 2 (**d**); GJ rigidity for case 1 (**e**) and case 2 (**f**).

As for the cases of the single-walled carbon nanotubes (SWCNTs) [40,41] and single-walled boron nitride nanotubes (SWBNNTs) [39], and for the phosphide NTs under study, the values of the tensile rigidity, EA, can be represented by a linear function of nanotube diameter, $D_{n}$ (see Figure 9a,b), and the values of bending, EI, and torsional, GJ, rigidities can be represented by a linear function of $D_{n}^{3}$ (see Figure 9c–f).

Similar to what was found in the authors’ previous work for the SWBNNTs [39], the straight lines in Figure 9a–f can be expressed as follows:

(33) $${\mathrm{EA}=\alpha }_{A13P}D_{n},$$

(34) $${\mathrm{EI}=\beta }_{A13P}D_{n}{}^{3},$$

(35) $${\mathrm{GJ}=\gamma }_{A13P}D_{n}{}^{3},$$

where ${\;\alpha }_{A13P}$, ${\;\beta }_{A13P}$ and $\gamma_{A13P}$ are the fitting parameters. The values of these parameters, obtained from the graphs in Figure 9a–f for single-walled phosphide NTs, are shown in Table 6.

The accuracy of the evaluation of the EA, EI and GJ rigidities values analytically estimated with Equations (33)–(35), respectively, was verified through the comparison with the values of the EA, EI and GJ rigidities calculated by Equations (30)–(32), respectively, using the data taken from FE analysis. The mean differences between the EA, EI and GJ values evaluated analytically and those obtained by the FE analysis are shown in Table 7. It can be seen from Table 7 that Equations (33)–(35) allow an accurate calculation of the three rigidities of SWBPNTs, SWAlPNTs, SWGaPNTs and SWInPNTs. The mean difference does not exceed 0.85%, which is the greatest value observed for the bending rigidity.

**Figure 10:**
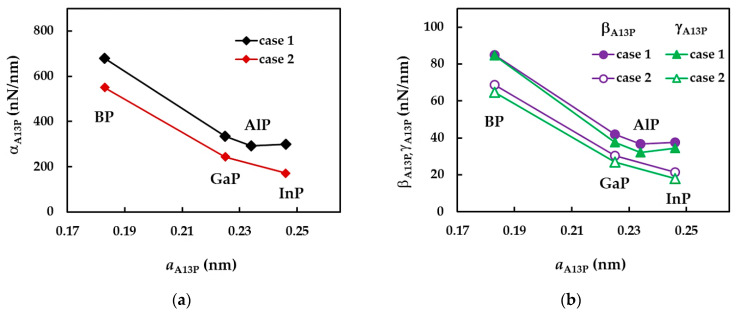
Evolutions of (**a**) $\alpha_{A13P}$, and (**b**) $\beta_{A13P}$ and $\gamma_{A13P}$ fitting parameters with the bond length, $a_{A13-P}$, for the two cases of input values in the numerical simulation of SWBPNTs, SWAlPNTs, SWGaPNTs and SWInPNTs.

In order to better comprehend the results of the tensile, EA, bending, EI, and torsional, GJ, rigidities presented in Figure 9, the evolutions of $\alpha_{A13P}$, $\beta_{A13P}$ and $\gamma_{A13P}$ fitting parameters with the bond length value, $a_{A13P}$ (see Table 2), considering the cases of SWBPNTs, SWAlPNTs, SWGaPNTs and SWInPNTs, are shown in Figure 10, for the two cases of input parameters. For case 1, all three fitting parameters decrease from SWBPNTs to SWAlPNTs, i.e., as the value of $a_{A13-P}$ increases, and then the values of $\alpha_{A13P}$, $\beta_{A13P}$ and $\gamma_{A13P}$ remain nearly unchanged when moving to SWInPNTs. For case 2, the fitting parameters $\alpha_{A13P}$, $\beta_{A13P}$ and $\gamma_{A13P}$ decrease with increasing bond length from SWBPNTs to SWInPNTs.

It should be noted that for case 1 of SWBPNTs, the ratio $\beta_{A13P}/\gamma_{A13P}$ is about 1, which means that the EI and GJ rigidities are nearly equal. The value of this ratio, $\beta_{A13P}/\gamma_{A13P}$ = 1.1, for SWBPNTs (case 2), SWAlPNTs, SWGaPNTs and SWInPNTs (case 1), indicates a certain difference between the values of bending, EI, and torsional, GJ, rigidities. This difference becomes higher for case 2 of SWInPNTs, for which $\beta_{A13P}/\gamma_{A13P}$ = 1.2.

### 3.2. Elastic Moduli of SWBPNTs, SWAlPNTs, SWGaPNTs and SWInPNTs

#### 3.2.1. Effect of Nanotube Wall Thickness on the Calculation of Elastic Moduli

As previously deduced for SWCNTs [40,41] and SWBNNTs [39], the Young’s and shear moduli of NTs structures can be calculated using the following expressions, respectively:

(36) $$E=\frac{\mathrm{EA}}{{\pi t}_{n}\sqrt{8\left(\frac{\mathrm{EI}}{\mathrm{EA}}\right){–t}_{n}^{2}}},$$

(37) $$G=\frac{\mathrm{GJ}}{{2\pi t}_{n}\left(\frac{\mathrm{EI}}{\mathrm{EA}}\right)\sqrt{8\left(\frac{\mathrm{EI}}{\mathrm{EA}}\right){–t}_{n}^{2}}},$$

where EA, EI and GJ are the tensile, bending and torsional rigidities, respectively, and $t_{n}$ is the nanotube wall thickness. The EA, EI and GJ rigidities can be calculated from the results of the FE analysis by Equations (30)–(32), respectively, or evaluated analytically using Equations (33)–(35), respectively. To date, regarding the NT wall thickness, no $t_{n}$ value, observed experimentally or calculated by theoretical approaches, has been reported for phosphide nanotubes. Furthermore, there are no reliable data indicating that the wall thickness value of phosphide NTs can be adopted equal to 0.34 nm (the graphite interlayer spacing).

**Figure 11:**
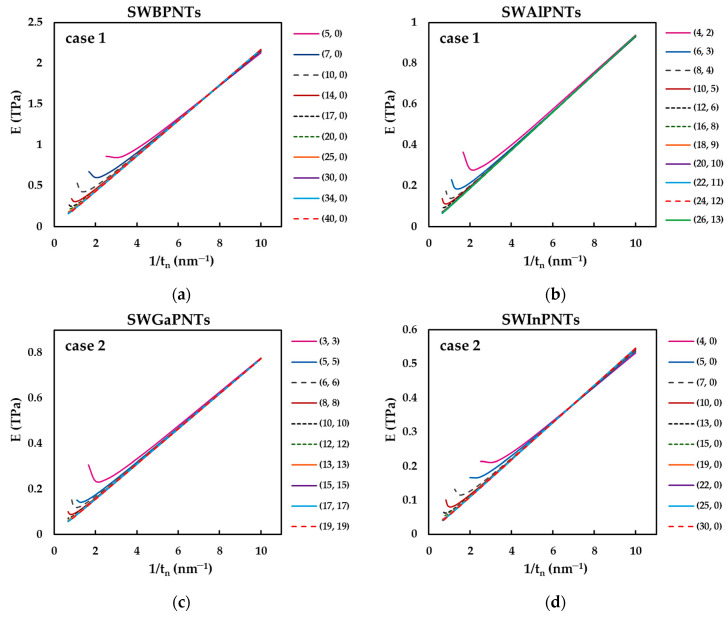
Evolution of the Young’s modulus, E, as a function of the inverse of the nanotube wall thickness, $t_{n}$, for: (**a**) (n, 0) SWBPNTs; (**b**) (n, m) SWAlPNTs; (**c**) (n, n) SWGaPNTs; and (**d**) (n, 0) SWInPNTs; (**a**,**b**) case 1, (**c**,**d**) case 2.

**Figure 12:**
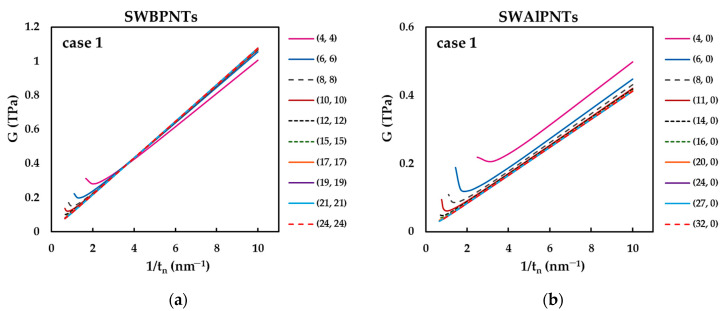

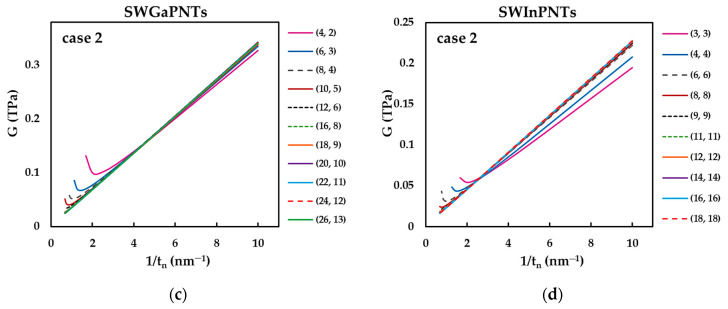
Evolution of the shear modulus, G, as a function of the inverse of the nanotube wall thickness, $t_{n}$, for: (**a**) (n, n) SWBPNTs; (**b**) (n, 0) SWAlPNTs; (**c**) (n, m) SWGaPNTs; and (**d**) (n, n) SWInPNTs; (**a**,**b**) case 1, (**c**,**d**) case 2.

For this reason, the Young’s, E, and shear, G, moduli determined by Equations (36) and (37), respectively, are plotted as a function of the inverse of the nanotube wall thickness, 1/$t_{n}$, (in the range $0.1\leq t_{n}\leq 1.5$ nm) for phosphide NTs selected from Table 5, as shown in Figure 11 (for E values) and Figure 12 (for G values). All three NT configurations, zigzag, chiral and armchair, and the two cases of input parameters are considered.

The evolutions of both the Young’s, E, and the shear, G, moduli as a function of the inverse nanotube wall thickness, 1/$t_{n}$, follow a quasi-linear trend for NTs with diameter $D_{n}\;≳$ 1.7 nm. For phosphide NTs with diameters $D_{n}\;≲$ 1.7 nm, the deviation from the quasi-linear trend occurs for high values of NTs wall thickness, when $t_{n}$ is in the range between the nanotube diameter and half the nanotube diameter, $D_{n}\;≲{\;t}_{n}\;≲D_{n}/2$. Thus, the smaller the value of $D_{n}$, the more noticeable is the deviation from the quasi-linearity of the evolution of the Young’s and shear moduli with 1/$t_{n}$. The SWBPNTs, SWAlPNTs, SWGaPNTs and SWInPNTs with diameters $D_{n}\;≲$ 1.7 nm and wall thickness in the range $D_{n}\;≲{\;t}_{n}\;≲D_{n}/2$ behave as solid cylinders instead of hollow tubes, which influences the results of elastic moduli.

#### 3.2.2. Surface Young’s Modulus of Phosphide Nanotubes

The lack of knowledge about the reliable wall thickness value has caused the studies, concerning evaluation of the elastic properties of the non-carbon nanotubes (N-CNTs), to mainly focus on determining the N-CNTs’ surface elastic moduli [42]. Regarding the elastic properties of the phosphide NTs, Kochaev [26] and Jiang and Guo [27], are the only authors who calculated the surface Young’s modulus of BP [27], AlP [26], GaP [26,27] and InPNTs [27]. Thus, to calculate the Young’s, E, and shear, G, moduli without the necessity of knowing the NT wall thickness and to facilitate the comparison with the literature results, a methodology for calculation of the surface Young’s, $E_{s}$, and shear, $G_{s}$, moduli, based on the linear evolutions of E and G moduli as a function of the inverse of the NT thickness, 1/$t_{n}$, is suggested.

As long as the wall thickness of phosphide nanotube is greater than half of its diameter, the Young’s and shear moduli become quasi-linear functions of the inverse wall thickness (see Figure 11 and Figure 12). The linear parts of the evolutions of E and G moduli with 1/$t_{n}$ can be described by expressions ${E=\alpha }_{1}\left(1/t_{n}\right)$ and ${G=\alpha }_{2}\left(1/t_{n}\right)$, respectively, where $\alpha_{1}$ and $\alpha_{2}$ are the slopes of the corresponding straight lines. Taking into account that the surface Young’s, $E_{s}$, modulus is the Young’s modulus, E, multiplied by the NT wall thickness, $E_{s}{=\mathrm{Et}}_{n}$, and, likewise, the surface shear modulus, $G_{s}$, is the shear modulus, G, multiplied by the NT wall thickness, $G_{s}{=\mathrm{Gt}}_{n}$, it can be written as

(38) $${\mathrm{Et}}_{n}{=\alpha }_{1}\;\Leftrightarrow {\;E}_{s}{=\alpha }_{1},$$

(39) $${\mathrm{Gt}}_{n}{=\alpha }_{2}\;\Leftrightarrow {\;G}_{s}{=\alpha }_{2}.$$

Thus, Equations (38) and (39) are the basis of the methodology to calculate the surface Young’s, $E_{s}$, and shear, $G_{s}$ moduli through the slope of the linear portion of the evolution of the corresponding elastic modulus (E or G) as a function of the inverse of the nanotube wall thickness.

In this context, the evolutions of the E and G moduli as a function of 1/$t_{n}$, as shown in the examples in Figure 11 and Figure 12, were plotted for all SWBPNTs, SWAlPNTs, SWGaPNTs and SWInPNTs in Table 5. Only the linear portions of the evolutions of the elastic moduli, with an R-squared value of at least 0.9997, which approximately corresponds to the NT wall thickness range $t_{n}\;≲{\;D}_{n}/3$, were considered for analysis. Then, to assess the surface Young’s, $E_{s}$, and shear, $G_{s}$, moduli, the slopes of straight lines were determined (see equations (38) and (39), respectively).

**Figure 13:**
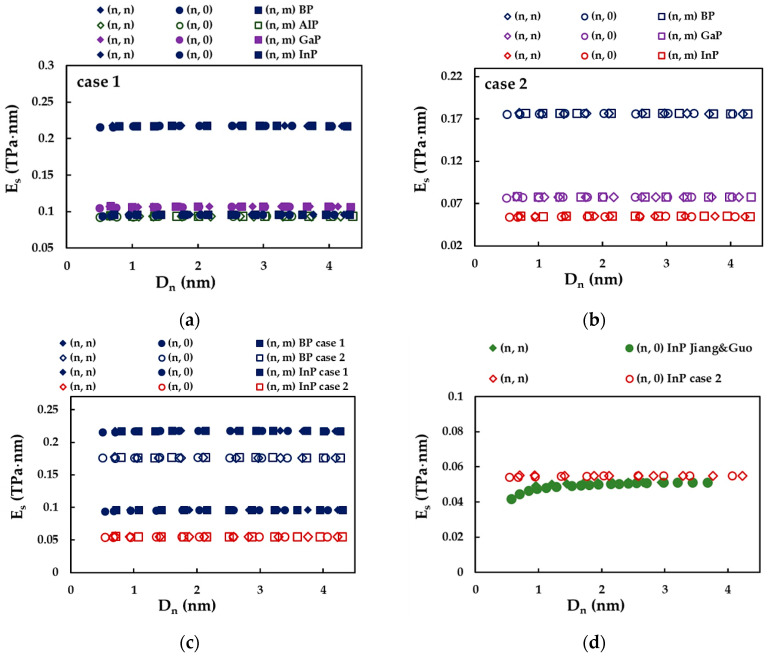
Evolution of surface Young’s modulus, $E_{s}$, as a function of the nanotube diameter, $D_{n}$: (**a**) for case 1 of SWBPNTs, SWAlPNTs, SWGaPNTs and SWInPNTs; (**b**) for case 2 of SWBPNTs, SWGaPNTs and SWInPNTs; (**c**) for case 1 and case 2 of SWBPNTs and SWInPNTs; and (**d**) comparison with the literature results [27].

Figure 13 shows the evolutions of $E_{s}$ with the diameter of the nanotubes, $D_{n}$, for SWBPNTs, SWAlPNTs, SWGaPNTs and SWInPNTs. The surface Young’s modulus is nearly stable over the entire diameter range of the NTs studied, regardless of the case of the input parameters, the NTs chirality and the first element (B, Al, Ga, In) of phosphide compound forming the nanotube. As can be seen in Figure 13a, for case 1 of input parameters, the SWBPNTs have the highest value of $E_{s}$ of about 0.218 TPa·nm, and the value of $E_{s}$ decreases approximately by half for SWAlPNTs ($E_{s}$ = 0.094 TPa·nm), SWGaPNTs ($E_{s}$ = 0.107 TPa·nm) and SWInPNTs ($E_{s}$ = 0.096 TPa·nm). For case 2 of input parameters, the surface Young’s modulus decreases from SWBPNTs to SWInPNTs, the $E_{s}$ value being approximately 2.2 and 3.2 times smaller for SWGaPNTs ($E_{s}$ = 0.078 TPa·nm) and SWInPNTs ($E_{s}$ = 0.055 TPa·nm), respectively, when compared with SWBPNTs ($E_{s}$ = 0.176 TPa·nm) (see Figure 13b). The values of $E_{s}$ calculated for case 1 are higher than those evaluated for case 2, whatever the NTs, as can be seen in Figure 13c for the SWBPNTs and SWInPNTs. Figure 13d compares the surface Young’s modulus, $E_{s}$, results obtained for case 2 of (n, n) and (n, 0) SWInPNTs with those available in the literature.

Among the literature results, the values of the surface Young’s modulus reported by Jiang and Guo [27] for (n, n) and (n, 0) SWInPNTs, which are in better agreement with the $E_{s}$ values calculated in the present study, were chosen for comparison. A mean difference of about 7.9% was observed for nanotubes with diameter $D_{n}\;≳$ 1.0 nm, when comparing the $E_{s}$ results by Jiang and Guo [27] with those obtained for case 2. Comprehensive comparison with the literature results appears to be difficult due to the scarcity of studies on the evaluation of the phosphide NTs’ surface Young’s modulus, but discrepancies were noticed in the reported $E_{s}$ values and trends. For example, in an ab initio simulation study, Kochaev [26] observed, for SWAlPNTs and SWGaPNTs, a considerable increase of the surface Young’s modulus, for (n, n) nanotubes when $D_{n}$ ≲ 1.2 nm and (n, 0) nanotubes when $D_{n}$ ≲ 0.9 nm, and then $E_{s}$ reaches the maximum, followed by a sharp decrease. An alternative trend reported in the literature for SWBPNTs, SWGaNTs and SWInPNTs [27] consists of a slight increase of the $E_{s}$ value when the nanotube diameter increases, and then $E_{s}$ becomes practically constant for NTs with diameters $D_{n}$ ≳ 0.7 nm (see Figure 13d for the case of SWInPNTs). Finally, the comparison with the available results was carried out as far as possible, as documented in Table 8.

In contrast to the reasonable agreement of the $E_{s}$ values for SWInPNTs assessed by Jiang and Guo [27] and those obtained for case 2 of the input parameters (see Figure 13c), less agreement is observed when the $E_{s}$ results reported in the same work [27] for SWBPNTs and SWGaPNTs are compared with the current results calculated for case 2. For SWBPNTs and SWGaPNTs with diameter $D_{n}\;≳$ 1.0 nm, the difference between the $E_{s}$ values by Jiang and Guo [27] and the current ones reaches ≈33% and ≈23%, respectively (see Table 8). These dissimilarities can be attributed to different calculation methods for the surface Young’s modulus and force field constants. Jiang and Guo [27] assessed $E_{s}$ employing closed-form analytical solutions based on the “stick-and-spring” model. To calculate the bond stretching, $k_{r}$, and bond bending, $k_{\theta }$, force constants for phosphide NTs, Jiang and Guo [27] modified the UFF method and used bond length values not equal to the present study. Moreover, they introduced a negative inversion force constant,$\;k_{\psi }$, without taking into account the dihedral torsion force constant,$\;k_{\phi }$. With regard to the results reported by Kochaev [26], the best agreement is observed when comparing with the current $E_{s}$ values for SWGaPNTs. The difference between the values of $E_{s}$ for case 1 and the maximum $E_{s}$ values evaluated by Kochaev [26] reaches ≈34% and ≈23% for (n, n) and (n, 0) GaP nanotubes, respectively (see Table 8).

**Figure 14:**
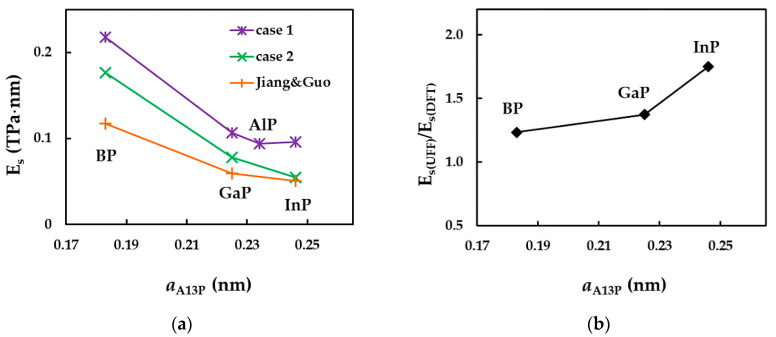
Evolutions of the: (**a**) surface Young’s modulus, $E_{s}$, for the two cases of input parameters, calculated in the present study, and evaluated by Jiang and Guo [27], and (**b**) ratio of the surface Young’s moduli, obtained for case 1 and 2, $E_{s(\mathrm{UFF})}/E_{s(\mathrm{DFT})}$, as a function of the bond length, $a_{A13-P}$.

To clarify the results shown in Figure 13, the surface Young’s modulus of the phosphide NTs is plotted as a function of the bond length, $a_{A13-P}$, for cases 1 and 2 of input parameters, in Figure 14a. The values of $E_{s}$ evaluated by Jiang and Guo [27] are also shown in Figure 14a. The evolution of the ratio of the surface Young’s moduli obtained for cases 1 and 2, $E_{s(\mathrm{UFF})}/E_{s(\mathrm{DFT})}$, with $a_{A13-P}$ is presented in Figure 14b.

For phosphide nanotubes, $E_{s}$ decreases with increasing bond length, $a_{A13-P}$, i.e., from SWBPNTs to SWInPNTs, except for case 1, for which the values of $E_{s}$ calculated for SWAlPNTs and SWInPNTs are nearly equal. Furthermore, the decreasing trend of the surface Young’s modulus with $a_{A13-P}$ can be easily established from the results of Jiang and Guo [27] (see Figure 14a).

Regarding the $E_{s}$ results calculated for case 1 (UFF) and case 2 (DFT+MM) of the input parameters for numerical simulation, the ratio of $E_{s(\mathrm{UFF})}/E_{s(\mathrm{DFT})}$ increases with the bond length of NTs, achieving the highest difference between the values of $E_{s}$ for the SWInPNTs. For these NTs, $E_{s}$ calculated for case 1 is 1.8 times larger than that for case 2.

#### 3.2.3. Surface Shear Modulus of Phosphide Nanotubes

**Figure 15:**
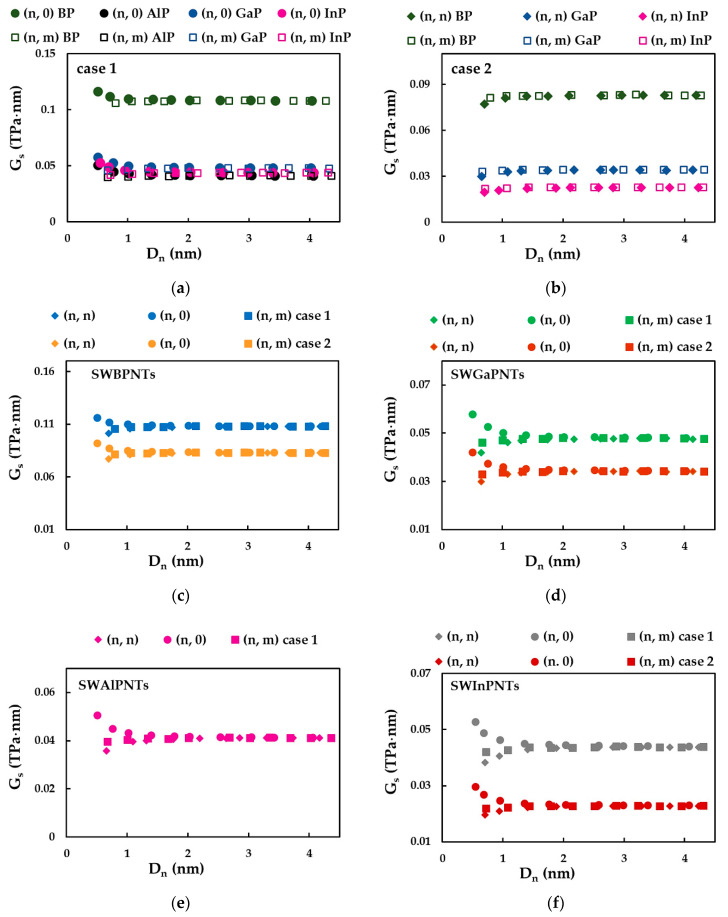
Evolution of the surface shear modulus, $G_{s}$, as a function of the nanotube diameter, $D_{n}$: (**a**) for case 1 of (n, 0) and (n, m) SWBPNTs, SWAlPNTs, SWGaPNTs and SWInPNTs; (**b**) for case 2 of (n, n) and (n, m) SWBPNTs, SWGaPNTs and SWInPNTs; (**c**) for SWBPNTs; (**d**) for SWGaNTs; (**e**) for SWAlPNTs; and (**f**) for SWInPNTs.

Figure 15 shows the evolutions of the surface shear modulus, $G_{s}$, calculated based on Figure 12 and Equation (39), as a function of the nanotube diameter, $D_{n}$, for SWBPNTs, SWAlPNTs, SWGaPNTs and SWInPNTs.

For small NT diameters, $D_{n}$, the surface shear modulus, $G_{s}$, decreases for zigzag (n, 0) NTs (Figure 15a) and increases for armchair (n, n) NTs (Figure 15b), being stable for high values of $D_{n}$, regardless of the NTs symmetry group. The surface shear modulus for chiral (n, m) NTs is nearly constant over the entire range of NT diameters and is equal to the converged average value of $G_{s}$, obtained for (n, n) and (n, 0) NTs (Figure 15a,b). For case 1, the SWBPNTs have the highest convergent average value of the surface shear modulus ($G_{s}$ = 0.108 TPa·nm) and $G_{s}$ decreases approximately 2.4 times for SWGaPNTs ($G_{s}$ = 0.048 TPa·nm), SWAlPNTs ($G_{s}$ = 0.041 TPa·nm) and SWInPNTs ($G_{s}$ = 0.044 TPa·nm). For case 2, the converged average value of $G_{s}$ decreases from SWBPNTs ($G_{s}$ = 0.083 TPa·nm) to SWGaPNTs ($G_{s}$ = 0.034 TPa·nm) and also for SWInPNTs, which have the lowest $G_{s}$ value of 0.023 TPa·nm. The value of $G_{s}$, of the three symmetry groups of phosphide NTs, for small diameters nanotubes depends on the chiral angle and decreases from zigzag (n, 0) NTs with Θ = 0° to chiral (n, m) NTs with Θ = 19.1°, and then to armchair (n, n) NTs with Θ = 30° (Figure 15c–f). It can be noted in Figure 15c–f that the greater the value of the bond length, $a_{A13-P}$ ,of the phosphide nanotube (see Table 2), the greater the value of the NT diameter, $D_{n}^{\mathrm{st}}$, for which the shear modulus becomes nearly constant, regardless of the NTs symmetry. The values of $D_{n}^{\mathrm{st}}$ are the same regardless of the input parameters case, although the values of $G_{s}$ calculated for case 1 are always higher than those for case 2.

**Figure 16:**
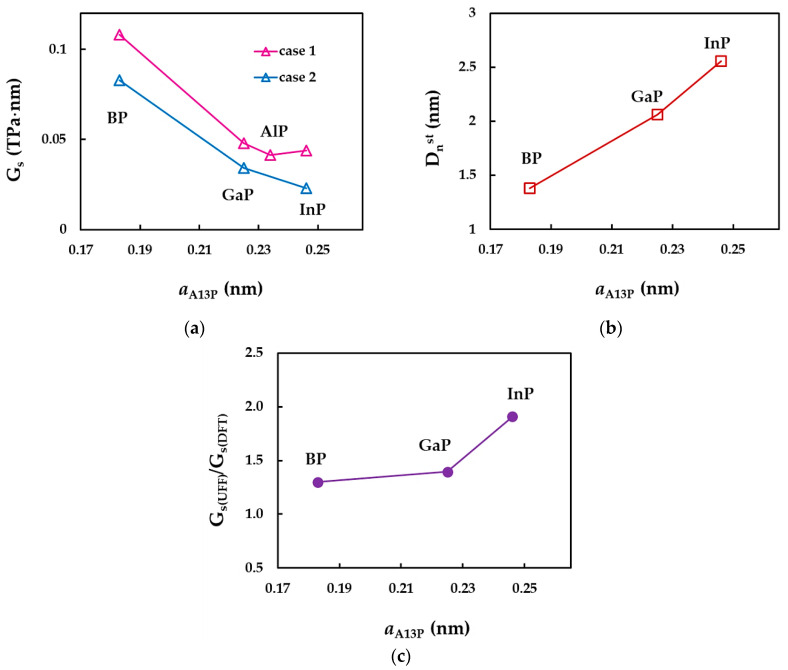
Evolutions of the: (**a**) surface shear modulus, $G_{s}$, for the two cases of input parameters, (**b**) NT diameter, $D_{n}^{\mathrm{st}}$, for which $G_{s}$ stabilizes, and (**c**) ratio of the surface shear moduli, obtained for case 1 and 2, $G_{s(\mathrm{UFF})}/G_{s(\mathrm{DFT})}$, as a function of the bond length, $a_{A13-P}$.

In order to understand better the results shown in Figure 15, the convergent average value of the surface shear modulus, $G_{s}$, the NT diameter, $D_{n}^{\mathrm{st}}$, for which the value of $G_{s}$ becomes stable, and the ratio between the surface shear moduli calculated for cases 1 and 2, $G_{s(\mathrm{UFF})}/G_{s(\mathrm{DFT})}$, were plotted in Figure 16 as a function of the NT bond length, $a_{A13-P}$.

As can be seen from Figure 16a, the decrease in the value of the surface shear modulus calculated for case 1 occurs when the bond length increases from 0.183 nm (SWBPNTs) to 0.225 nm (SWGaPNTs). Nearby are the SWAlPNTs and SWInPNTs nanotubes, for which the values of $G_{s}$ are close to each other and to those evaluated for SWGaPNTs. For case 2, the $G_{s}$ value decreases with increasing of $a_{A13-P}$. On the contrary, the diameter, $D_{n}^{\mathrm{st}}$, increases with increasing the bond length (see Figure 16b). With regard to the ratio $G_{s(\mathrm{UFF})}/G_{s(\mathrm{DFT})}$, the values of $G_{s}$ obtained for case 1 (UFF) is 1.3, 1.4 and 1.9 times bigger than those calculated for case 2 (DFT + MM) for SWBPNTs, SWGaPNTs and SWInPNTs, respectively (see Figure 16c).

It is worth noting that, similarly to the surface Young’s modulus, the shear modulus of phosphide NTs is also sensitive to the input parameters for numerical simulation, which in turn depend on the bond length and the force field constants.

Since the phosphide NTs have a potential application in the NT-based devices and hybrid nanostructures, where they can be combined with carbon or other non-carbon NTs, the surface Young’s and shear moduli of the SWBPNTs, SWAlPNTs, SWGaPNTs and SWInPNTs were compared with respective surface elastic moduli of the SWCNTs and SWBNNTs in Table 9. $E_{s}$ and $G_{s}$ of carbon and boron-nitride NTs were calculated from the results of the authors’ previous work [39], using the expressions $E_{s}{=\mathrm{Et}}_{n}$ and $G_{s}{=\mathrm{Gt}}_{n}$, respectively, and taking into account that the value of the nanotube wall thickness $t_{n}$ = 0.34 nm, for both classes of NTs.

It can be concluded from Table 9 that SWBPNTs and, particularly, SWAlPNTs, SWGaPNTs and SWInPNTs have weak mechanical properties when compared with SWCNTs and SWBNNTs. Thus, when designing and constructing NT-based devices and hybrid nanostructures, it is desirable to combine the phosphide NTs, with low mechanical strength, with CNTs or N-CNTs, with high mechanical strength.

### 3.3. Poisson’s Ratio of SWBPNTs, SWAlPNTs, SWGaPNTs and SWInPNTs

The Poisson’s ratio of the phosphide NTs can be calculated, assuming the isotropy condition and considering Equations (36) and (37), by the following expression:

(40) $$\nu =\frac{E}{2G}\;–\;1=\frac{\mathrm{EI}}{\mathrm{GJ}}\;–\;1,$$

where EI and GJ are bending and torsional rigidities, respectively. The EI and GJ rigidities can be calculated either from the results of FE analysis by Equations (31) and (32), respectively, or analytically using Equations (34) and (35), respectively.

Combining Equation (40) with relationships (34) and (35), the Poisson’s ratio can be expressed by an equation independent of the NT diameter, through the fitting parameters $\beta_{A13P}$ and $\gamma_{A13P}$, as follows:

(41) $$\nu =\frac{\beta_{A13P}}{\gamma_{A13P}}\;–\;1.$$

**Figure 17:**
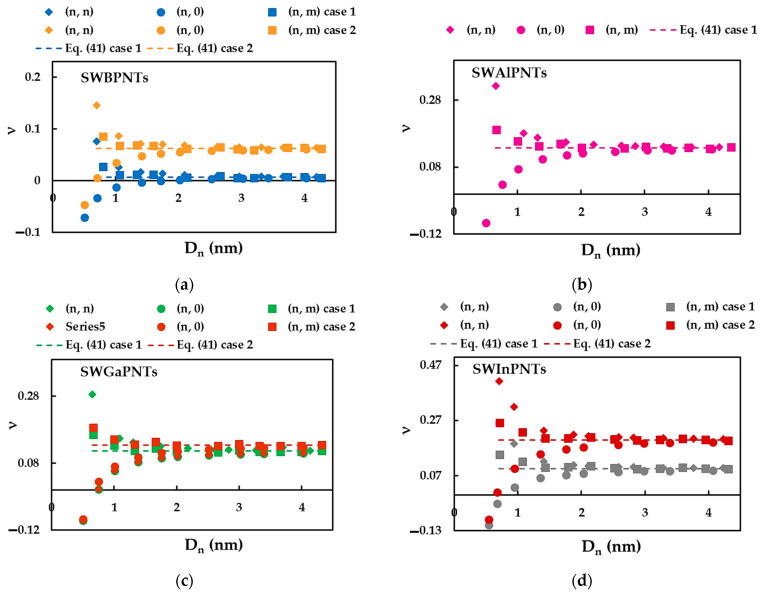
Evolution of the Poisson’s ratio, ν, as a function of the nanotube diameter, $D_{n}$, for: (**a**) SWBPNTs, (**b**) SWAlPNTs, (**c**) SWGaPNTs and (**d**) SWInPNTs.

Figure 17 shows the evolution of the Poisson’s ratio, ν, calculated by Equation (40), with the NT diameter, $D_{n}$, for SWBPNTs, SWAlPNTs, SWGaPNTs and SWInPNTs in Table 5. The two cases of the analyzed input parameters are considered. The values of ν calculated by Equation (41), which is independent of $D_{n}$, are also presented in Figure 17. The Poisson’s ratio evaluated for case 2 is higher than that for case 1. For (n, n), (n, 0) and (n, m) phosphide NTs with high diameters, the Poisson’s ratio converges to the constant value calculated by Equation (41). The greater the value of the bond length, $a_{A13-P}$, the greater the value of the nanotube diameter, $D_{n}^{\mathrm{st}}$, for which ν becomes stable (see Figure 17). The value of $D_{n}^{\mathrm{st}}$ does not depend on the case of input parameters used for numerical simulations.

For phosphide NT diameter lower than $D_{n}^{\mathrm{st}}$, the Poisson’s ratio decreases for (n, n) armchair and (n, m) chiral NTs, but for (n, 0) zigzag NTs, the ν value increases. In addition, the (n, 0) phosphide NTs with small diameters $D_{n}$ < 1.0 nm have a negative Poisson’s ratio (demonstrate an auxetic behavior). It can also be seen from Figure 17 that for NTs with a diameter smaller than the diameter $D_{n}^{\mathrm{st}}$, the Poisson’s ratio noticeably depends on the chiral angle and is greater for (n, n) NTs (Θ = 30°) and smaller for (n, 0) NTs (Θ = 0°). This effect is particularly evident for the small phosphide NT diameters, $D_{n}$ ≤ 1.0 nm. A similar dependence of the value of ν on the NT chiral angle was reported for the SWBNNTs with diameter below 1.5 nm [39]. This result was explained by the fact that the ratio between bending and torsion rigidities, EI/GJ, did not have a constant value for $D_{n}$< 1.5 nm.

**Figure 18:**
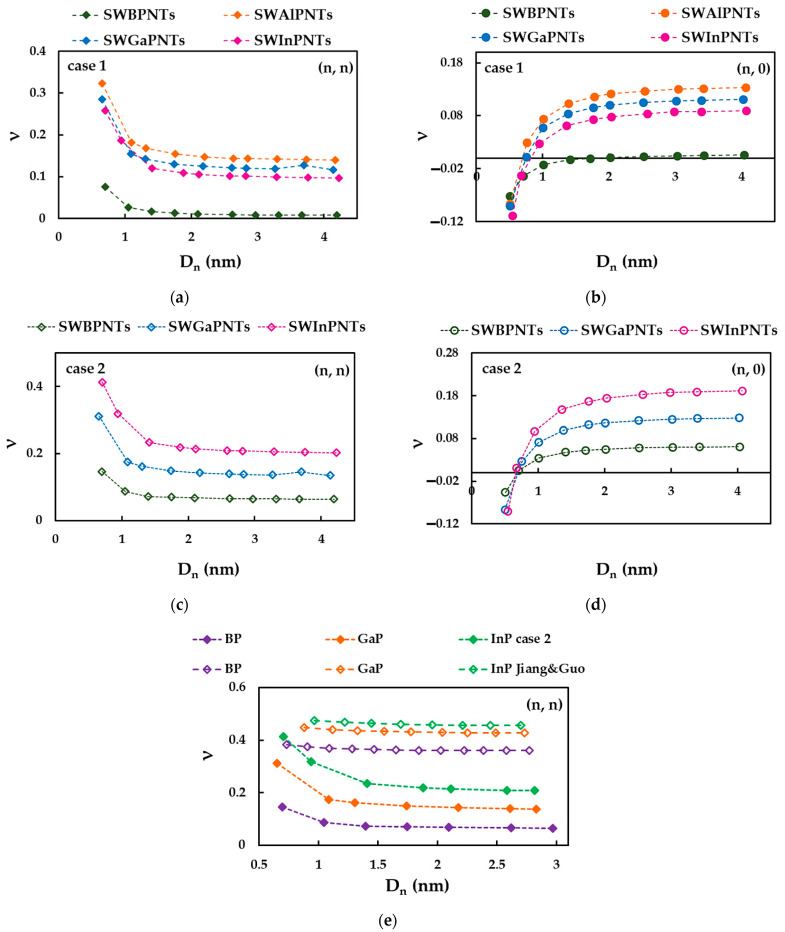
Evolution of the Poisson’s ratio, ν, as a function of the nanotube diameter, $D_{n}$, for: (**a**) (n, n) SWBPNTs, SWAlPNTs, SWGaPNTs and SWInPNTs, case 1, (**b**) (n, 0) SWBPNTs, SWAlPNTs, SWGaPNTs and SWInPNTs, case 1, (**c**) (n, n) SWBPNTs, SWGaPNTs and SWInPNTs, case 2, (**d**) (n, 0) SWBPNTs, SWGaPNTs and SWInPNTs, case 2, and (**e**) (n, n) SWBPNTs, SWGaPNTs and SWInPNTs, case 2 and Jiang and Guo [27].

To analyze the influence of the input parameters on the Poisson’s ratio results, the evolutions of the Poisson’s ratio, ν, as a function of the NT diameter, $D_{n}$, for cases 1 and 2 of (n, 0) and (n, n) SWBPNTs, SWAlPNTs, SWGaPNTs and SWInPNTs are plotted in Figure 18. The available literature results for (n, n) SWBPNTs, SWGaPNTs and SWInPNTs are also shown (Figure 18e). As can be seen in Figure 18a,b, for case 1 of the phosphide NTs, the convergent average value of ν increases from SWBPNTs (ν = 0.01) to SWAlPNTs (ν = 0.14), and then decreases for SWInPNTs (ν = 0.09). For case 2, the Poisson’s ratio grows from 0.06 for SWBPNTs to 0.20 for SWInPNTs (see Figure 18c,d). The values of ν obtained for cases 1 and 2 of SWGaPNTs are close and equal to 0.12 and 0.13, respectively.

Although the Poisson’s ratio values evaluated by Jiang and Guo [27] for SWBPNTs, SWGaPNTs and SWInPNTs are 83%, 69% and 56% higher, respectively, than those calculated in the present study for case 2, it is possible to compare the trends of the evolutions of ν as a function of NT diameter, $D_{n}$, as shown in Figure 18d. Jiang and Guo [27] found that for (n, n) and (n, 0) phosphide NTs, the Poisson’s ratio decreases with increasing $D_{n}$, and then the ν value converges to an approximately constant value. This trend is in agreement with the current trend of evolution of ν with NT diameter for (n, n) phosphide NTs, although, the decreasing rate is slower for the ν evolution reported by Jiang and Guo [27].

**Figure 19:**
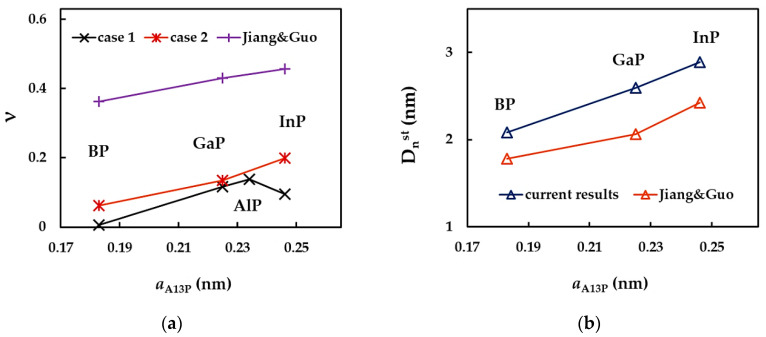
Evolutions of the: (**a**) Poisson’s ratio, ν, and (**b**) NT diameter, $D_{n}^{\mathrm{st}}$, for which the value of ν becomes stable, as a function of the bond length, $a_{A13-P}$.

To clarify the results shown in Figure 17 and Figure 18, the convergent average value of the Poisson’s ratio, ν, and the NT diameter, $D_{n}^{\mathrm{st}}$, for which the value of ν becomes stable, are plotted as a function of the NT bond length, $a_{A13-P}$ in Figure 19. The values of ν evaluated by Jiang and Guo [27] are presented in both plots for comparison purposes.

The Poisson’s ratio, ν, obtained for case 1 of phosphide NTs increases with the increasing of the $a_{A13-P}$ value up to 0.234 (SWAlPNTs), and then ν drops after $a_{A13-P}$ = 0.246 nm (SWInPNTs). For case 2, the value of ν increases with increasing bond length, which is in a good agreement with the results of Jiang and Guo [27] (see Figure 19a). Moreover, the same good agreement is found between trends of $D_{n}^{\mathrm{st}}$ values, obtained in the present work and reported by Jiang and Guo [27]. In both studies, it is shown that the greater the bond length, $a_{A13-P}$, the greater the NT diameter, $D_{n}^{\mathrm{st}}$, for which ν becomes stable (see Figure 19b). It is worth mentioning that the $D_{n}^{\mathrm{st}}$ values obtained in the present study and reported in the work of Jiang and Guo [27] are close.

The current Poisson’s ratio results for (n, n) and (n, 0) phosphide NTs are summarized in Table 10 together with the values of ν available in the literature.

It can be seen from Table 10 that the Poisson’s ratio results are scarce so far and the existing ν values show large scattering, regardless of the modelling and calculation approaches used for this end. Analyzing current Poisson’s ratio results and those reported by Jiang and Guo [27], it can be concluded that, similar to the surface elastic moduli, the ν values are sensitive to the values of bond length and force field constants.

## 4. Conclusions

The elastic properties, comprising the three rigidities, tensile, bending and torsional, the surface Young’s and shear moduli and the Poisson’s ratio of SWBPNTs, SWAlPNTs, SWGaPNTs and SWInPNTs were evaluated resorting to a numerical simulation study, based on the NCM/MSM approach. The main conclusions of the present study are indicated below.

The force field constants, required for calculating the input parameters for numerical simulation, were computed for BP, AlP, GaP and InP nanostructures, using two approaches, which resulted in two input sets.

Equations describing the relationship between each of the three rigidities and the nanotube diameter were obtained for SWBPNTs, SWAlPNTs, SWGaPNTs and SWInPNTs, and the fitting parameters for the Equations (30)–(32) were calculated for two sets of input parameters for numerical simulation. This allowed to expand, to phosphide nanotubes, the previously established method, allowing the calculation of tensile, bending and torsional rigidities without resorting to numerical simulation.

A robust methodology was proposed to assess the surface Young’s and shear moduli of the SWBPNTs, SWAlPNTs, SWGaPNTs and SWInPNTs, based on the Young’s and shear moduli evolutions as a function of the inverse nanotube thickness. It is expected that this methodology will be useful to evaluate the surface elastic moduli of the N-CNTs, for which the exact value of the nanotube wall thickness is unknown.

The tensile, bending and torsional rigidities, the surface Young’s and shear moduli, and the Poisson’s ratio of SWBPNTs, SWAlPNTs, SWGaPNTs and SWInPNTs are sensitive to the bond length and force field constants of the diatomic phosphide nanostructures.

The results obtained provide a substantial contribution to a benchmark with regard to the determination of the elastic properties of the phosphide nanotubes by numerical and analytical methods.

## Figures and Tables

**Table 1:** Bond length values of phosphide nanostructures available in the literature.

	BP	AlP	GaP	InP
$a_{A13-P}$, nm	0.183 [28] 0.193 [29]	0.234 [17] 0.240 [26]	0.220 [26] 0.225 [28] 0.229 [25] 0.236 [29]	0.246 [28] 0.256 [29]

**Table 2:** Effective charges of atoms [31], and bond length, surface Young’s modulus and Poisson’s ratio, obtained from DFT computations [28], for phosphide nanostructures.

Compound	Atom 1	Atom 2	$Z_{1}^{*}$	$Z_{2}^{*}$	$a_{A13-P}$, nm	E_s_*,* nN/nm [28]	ν [28]
			Charge [31]	Charge [31]			
BP	B	P	1.755	2.863	0.183 [28]	135	0.28
AlP	Al		1.792		0.234 [17]	–	–
GaP	Ga		1.821		0.225 [28]	59	0.35
InP	In		2.070		0.246 [28]	39	0.43

**Table 3:** $k_{r}$, $k_{\theta }$ and $k_{\tau }$ force field constants for phosphide nanostructures.

Compound	Case ^1^	$k_{r},\;\mathrm{nN}/\mathrm{nm}$	$k_{\theta 1},\;\mathrm{nN}\cdot \mathrm{nm}/{\mathrm{rad}}^{2}$	$k_{\theta 2},\;\mathrm{nN}\cdot \mathrm{nm}/{\mathrm{rad}}^{2}$	$k_{\tau },\;\mathrm{nN}\cdot \mathrm{nm}/{\mathrm{rad}}^{2}$
BP	1	379	1.486	0.558	0.625
	2	325	1.031	0.387	
AlP	1	185	0.711	0.278	
	2	–	–	–	
GaP	1	211	0.853	0.345	
	2	157	0.599	0.242	
InP	1	184	0.852	0.446	
	2	119	0.391	0.204	

^1^ Case 1 corresponds to the UFF calculation method, and case 2 to DFT+MM.

**Table 4:** Geometrical and elastic properties of the beam element (input values for FE simulations).

Compound	Case ^1^	*l*, nm [17,28]	d, nm Equation (26)	E_b_, GPa Equation (27)	G_b_, GPa Equation (28)	ν_b_ Equation (29)
BP	1	0.183	0.2078	2042	624	0.21
	2		0.1869	2165	954	0.28
GaP	1	0.225	0.2130	1335	696	0.33
	2		0.2069	1052	782	0.35
AlP	1	0.234	0.2069	1287	813	0.38
	2		-	-	-	-
InP	1	0.246	0.2377	1019	491	0.32
	2		0.2004	924	722	0.43

^1^ Case 1 corresponds to the UFF calculation method, and case 2 to DFT+MM.

**Table 5:** Chiral indices and diameters of the SWBPNTs, SWAlPNTs, SWGaPNTs and SWInPNTs.

NT type	SWBPNTs		SWAlPNTs		SWGaPNTs		SWInPNTs	
	(n, m)	D_n_, nm	(n, m)	D_n_, nm ^1^	(n, m)	D_n_, nm ^1^	(n, m)	D_n_, nm
armchair (n, n), Θ = 30°	(4, 4)	0.699	(3, 3)	0.659	(3, 3)	0.653	(3, 3)	0.705
	(6, 6)	1.049	(5, 5)	1.098	(5, 5)	1.089	(4, 4)	0.940
	(8, 8)	1.398	(6, 6)	1.318	(6, 6)	1.306	(6, 6)	1.409
	(10, 10)	1.748	(8, 8)	1.757	(8, 8)	1.742	(8, 8)	1.879
	(12, 12)	2.097	(10, 10)	2.196	(10, 10)	2.177	(9, 9)	2.114
	(15, 15)	2.621	(12, 12)	2.636	(12, 12)	2.613	(11, 11)	2.584
	(17, 17)	2.971	(13, 13)	2.855	(13, 13)	2.830	(12, 12)	2.819
	(19, 19)	3.320	(15, 15)	3.295	(15, 15)	3.266	(14, 14)	3.289
	(21, 21)	3.670	(17, 17)	3.734	(17, 17)	3.701	(16, 16)	3.759
	(24, 24)	4.194	(19, 19)	4.173	(19, 19)	4.137	(18, 18)	4.228
zigzag (n, 0), Θ = 0°	(5, 0)	0.504	(4, 0)	0.507	(4, 0)	0.503	(4, 0)	0.543
	(7, 0)	0.706	(6, 0)	0.761	(6, 0)	0.754	(5, 0)	0.678
	(10, 0)	1.009	(8, 0)	1.014	(8, 0)	1.006	(7, 0)	0.949
	(14, 0)	1.413	(11, 0)	1.395	(11, 0)	1.383	(10, 0)	1.356
	(17, 0)	1.715	(14, 0)	1.775	(14, 0)	1.760	(13, 0)	1.763
	(20, 0)	2.018	(16, 0)	2.029	(16, 0)	2.011	(15, 0)	2.034
	(25, 0)	2.522	(20, 0)	2.536	(20, 0)	2.514	(19, 0)	2.577
	(30, 0)	3.027	(24, 0)	3.043	(24, 0)	3.017	(22, 0)	2.984
	(34, 0)	3.430	(27, 0)	3.424	(27, 0)	3.394	(25, 0)	3.391
	(40, 0)	4.036	(32, 0)	4.058	(32, 0)	4.022	(30, 0)	4.069
chiral (n, m), Θ = 19.1°	(6, 3)	0.801	(4, 2)	0.671	(4, 2)	0.665	(4, 2)	0.718
	(8, 4)	1.068	(6, 3)	1.006	(6, 3)	0.998	(6, 3)	1.077
	(10, 5)	1.335	(8, 4)	1.342	(8, 4)	1.330	(8, 4)	1.435
	(12, 6)	1.602	(10, 5)	1.677	(10, 5)	1.663	(10, 5)	1.794
	(16, 8)	2.136	(12, 6)	2.013	(12, 6)	1.995	(12, 6)	2.153
	(20, 10)	2.669	(16, 8)	2.684	(16, 8)	2.661	(14, 7)	2.512
	(22, 11)	2.936	(18, 9)	3.019	(18, 9)	2.993	(16, 8)	2.871
	(24, 12)	3.203	(20, 10)	3.355	(20, 10)	3.326	(18, 9)	3.230
	(28, 14)	3.737	(22, 11)	3.690	(22, 11)	3.658	(20, 10)	3.588
	(30, 15)	4.004	(24, 12)	4.026	(24, 12)	3.991	(22, 11)	3.947
	(32, 16)	4.271	(26, 13)	4.361	(26, 13)	4.324	(24, 12)	4.306

^1^ Diameters, $D_{n}$, of SWAlPNTs and SWGaPNTs were calculated assuming Al-P length $a_{\mathrm{Al}-P}$ = 0.230 nm and Ga-P length $a_{\mathrm{Ga}-P}$ = 0.228 nm as defined by software Nanotube Modeler©.

**Table 6:** Fitting parameters ${\;\alpha }_{A13P}$, ${\;\beta }_{A13P}$ and $\gamma_{A13P}$ for phosphide nanotubes.

NTs	Case	${\;\alpha }_{A13P},\;\mathrm{nN}/\mathrm{nm}$	${\;\beta }_{A13P},\;\mathrm{nN}/\mathrm{nm}$	$\gamma_{A13P},\;\mathrm{nN}/\mathrm{nm}$
BP	1	680.40	84.99	84.44
	2	550.96	68.80	64.75
AlP	1	292.99	36.60	32.17
	2	–	–	–
GaP	1	334.07	41.78	37.43
	2	243.15	30.40	26.81
InP	1	300.5	37.54	34.29
	2	171.35	21.39	17.84

**Table 7:** Mean difference between the rigidity values of SWBPNTs, SWAlPNTs, SWGaPNTs and SWInPNTs evaluated with Equations (33)–(35) and the corresponding values obtained from the FE analysis.

NTs	Case	Mean Difference, %		
		EA, nN	EI, nN·nm^2^	GJ, nN·nm^2^
BP	1	0.34	0.60	0.37
	2	0.25	0.65	0.43
AlP	1	0.53	0.83	0.51
	2	–	–	–
GaP	1	0.38	0.82	0.55
	2	0.40	0.85	0.52
InP	1	0.39	0.77	0.43
	2	0.39	0.82	0.50

**Table 8:** Comparison of the current surface Young’s modulus results for phosphide nanotubes with those reported in the literature.

Reference	NT Type	E_s_, TPa				Comments
		SWBPNTs	SWAlPNTs	SWGaPNTs	SWInPNTs	
Kochaev [26]	(n, n)	-	0.228	0.161	-	maximum value
	(n, 0)		0.208	0.139		
	(n, n)		0.050	0.050		minimum value
	(n, 0)		0.072	0.025		
Jiang and Guo [27]	(n, n)	0.118	-	0.060	0.051	converged average value
	(n, 0)	0.117		0.059	0.051	
Present study	(n, n)	0.218 ^1^	0.094 ^1^	0.107 ^1^	0.096 ^1^	average value
		0.176 ^2^	-	0.078 ^2^	0.055 ^2^	
	(n, 0)	0.218 ^1^	0.094 ^1^	0.107 ^1^	0.096 ^1^	
		0.176 ^2^	-	0.078 ^2^	0.055 ^2^	

^1^ For case 1 (UFF); ^2^ For case 2 (DFT+MM).

**Table 9:** Surface elastic moduli of carbon, boron nitride and phosphide single-walled nanotubes.

Elastic Moduli	SWCNTs	SWBNNTs	SWBPNTs	SWAlPNTs	SWGaPNTs	SWInPNTs
$E_{s}$, TPa·nm	0.361 [39,40]	0.335 [39]	0.218 ^1^	0.094 ^1^	0.107 ^1^	0.096 ^1^
			0.176 ^2^		0.078 ^2^	0.055 ^2^
$G_{s}$, TPa·nm	0.171 [39,41]	0.165 [39]	0.108 ^1^	0.041 ^1^	0.048 ^1^	0.044 ^1^
			0.083 ^2^		0.034 ^2^	0.023 ^2^

^1^ For case 1 (UFF); ^2^ For case 2 (DFT+MM).

**Table 10:** Comparison of the current Poisson’s ratio results for phosphide nanotubes with those reported in the literature.

Reference	NT Type	ν				Comments
		SWBPNTs	SWAlPNTs	SWGaPNTs	SWInPNTs	
Kochaev [26]	(10, 10)	-	0.51	0.51	-	-
	(10, 0)		0.51	0.52		
Jiang and Guo [27]	(n, n)	0.36	-	0.43	0.46	converged average value
	(n, 0)	0.36		0.44	0.46	
Present study	(n, n)	0.01 ^1^	0.14 ^1^	0.12 ^1^	0.10 ^1^	converged average value
		0.06 ^2^	-	0.14 ^2^	0.055 ^2^	
	(n, 0)	0.01 ^1^	0.13 ^1^	0.11 ^1^	0.09 ^1^	
		0.06 ^2^	-	0.12 ^2^	0.19 ^2^	

^1^ For case 1 (UFF); ^2^ For case 2 (DFT+MM).

## Data Availability

The data presented in this study are available on request from the corresponding author after obtaining permission of an authorized person.
